# Cellular Immunotherapy for Cervical Cancer: Next Therapeutics Frontiers

**DOI:** 10.32604/or.2026.084736

**Published:** 2026-08-13

**Authors:** Danning Zhao, Qin Liu

**Affiliations:** Department of Obstetrics and Gynecology, Gusu School of Nanjing Medical University, the First People’s Hospital of Kunshan, Suzhou, China

**Keywords:** Cell therapy, cervical cancer, immunotherapy, tumor-infiltrating lymphocytes, T-cell receptor (TCR)-engineered T cells, chimeric antigen receptor (CAR)-T cells, clinical trials

## Abstract

Cervical cancer, particularly its advanced stages, requires novel therapeutic paradigms. Cellular immunotherapy exploits the constitutive expression of HPV E6/E7 oncoproteins as near-ideal tumor-specific antigens. This review systematically evaluates four principal platforms under investigation: tumor-infiltrating lymphocytes (TILs), TCR-engineered T cells, CAR-T cells, and CAR-NK cells. We critically analyze the preclinical rationale, clinical trial landscape, safety considerations, and manufacturing challenges for each modality. TIL therapy has achieved durable complete responses and an FDA Breakthrough Therapy designation. TCR-T cells enable precise targeting of intracellular viral epitopes but are HLA-restricted. CAR-T cells offer potent, MHC-independent recognition, yet face on-target/off-tumor toxicity and a suppressive tumor microenvironment. CAR-NK cells present a favorable safety profile and off-the-shelf potential. We conclude that the future of this field lies in rational combination strategies (e.g., with immune checkpoint blockade) and next-generation engineering (e.g., armored CARs, logic gates, allogeneic platforms) to overcome manufacturing complexity, toxicity, and high costs. Overcoming these barriers is essential to extend these therapies to resource-limited settings where the burden of cervical cancer is highest.

## 1 Introduction

Cervical cancer persists as a striking example of global oncological inequity and remains the fourth most common malignancy and fourth leading cause of cancer-related mortality among women worldwide [1,2]. According to GLOBOCAN 2022 estimates, the disease accounted for approximately 661,000 incident cases and 348,000 deaths globally, with a profound disparity in outcomes, 94% of these deaths occur in low- and middle-income countries, a trend attributable principally to inadequate access to prophylactic vaccination and high-quality screening infrastructure [3]. China bears a particularly substantial disease burden, with an estimated 150,700 new diagnoses and 55,700 deaths annually [4,5,6]. Notably, despite incremental improvements in cytology and HPV-based screening coverage, incidence rates in China exhibit a concerning upward trajectory among younger demographic cohorts [7]. The aetiological primacy of persistent infection with high-risk human papillomavirus (HPV) is unequivocal; HPV16 and HPV18 collectively account for over 70% of cervical squamous cell carcinomas globally. Within the general female population in China, the pooled prevalence of high-risk HPV infection approaches 17.70%, indicating a considerable reservoir of premalignant cervical intraepithelial neoplasia and underscoring the exigent need for expanded vaccine dissemination [8,9,10]. Therapeutic stratification is governed primarily by the International.

Federation of Gynecology and Obstetrics (FIGO) staging system [11]. Early-stage disease (FIGO IA-IB2) is typically managed via radical hysterectomy with pelvic lymphadenectomy or, in selected patients desiring fertility preservation, radical trachelectomy; 5-year overall survival in this cohort exceeds 90% [12,13]. For locally advanced disease (FIGO IB3–IVA), the cornerstone of management is definitive concurrent chemoradiotherapy, comprising external beam radiotherapy with concurrent cisplatin-based radiosensitization followed by intracavitary brachytherapy [14]. Recent landmark trials have established incremental gains in progression-free survival with the adjuvant incorporation of immune checkpoint inhibitors following completion of chemoradiation [15,16]. In the metastatic or recurrent setting, first-line systemic therapy comprises platinum-based doublet chemotherapy combined with bevacizumab, or the integration of pembrolizumab for tumors exhibiting PD-L1 positivity [17].

Despite these advances, outcomes for advanced-stage disease remain dismal. Treatment failure rates in locally advanced cervical cancer approach 30–40%, and longitudinal cohort analyses document a 10-year cumulative recurrence rate of 44% for stage III disease, escalating precipitously to 85% in stage IVA disease [18]. Moreover, survivors contend with substantial late-effect morbidity, including chronic radiation toxicity and premature surgical menopause. Although the approval of antibody-drug conjugates (ADCs), most notably tisotumab vedotin, has modestly extended the armamentarium for recurrent and metastatic disease, durable responses remain elusive and primary resistance constitutes a persistent biological barrier [19,20,21]. Thus, while population-level prevention and early detection have yielded remarkable successes, advanced-stage cervical cancer continues to circumvent conventional therapeutic paradigms. Addressing this recalcitrant malignancy will necessitate a concerted, multidisciplinary endeavor to develop next-generation agents and platform technologies capable of subverting intrinsic resistance mechanisms and ameliorating outcomes for the most vulnerable patient populations [22].

Despite the therapeutic advancements in cervical cancer, the unique immune landscape of this malignancy presents formidable barriers to durable therapeutic efficacy [23]. Central to this challenge is the multifaceted immune evasion program orchestrated by HPV oncoproteins E5, E6 and E7, which collectively dismantle effective antitumor immunity [24]. E5 downregulates surface MHC class I expression, thereby attenuating CD8^+^ T-cell recognition of HPV-derived epitopes. E6 and E7 further impair dendritic cell (DC) maturation and antigen presentation, disrupt interferon signaling, and activate immune checkpoint pathways, including the PD-L1/PD-1 axis [25]. This oncoprotein-driven immunosuppression is compounded by a profoundly hostile tumor microenvironment (TME) characterized by the recruitment and expansion of regulatory T cells (Tregs), myeloid-derived suppressor cells (MDSCs), and tumor-associated macrophages (TAMs) [26]. Notably, tertiary lymphoid structures within cervical tumors exhibit disproportionately high Treg and polymorphonuclear MDSC densities relative to other HPV-associated malignancies, and this infiltration strongly correlates with the absence of HPV-specific CD8^+^ T cells and diminished TCRζ chain expression [27]. Additional layers of immune subversion include hypoxia-induced attenuation of epitope presentation, IDO1-mediated tryptophan catabolism, and a Th2-skewed cytokine milieu enriched in interleukin-10 (IL-10) and TGF-β [28,29]. Collectively, these mechanisms conspire to foster T-cell exhaustion, limit effector cell trafficking, and confer primary and acquired resistance to immune checkpoint blockade [30,31]. Consequently, overcoming this recalcitrant immunosuppressive architecture will require next-generation strategies that simultaneously target viral oncoprotein function, reprogram the TME, and restore functional HPV-specific T-cell immunity.

Cellular immunotherapy has emerged as a transformative paradigm in oncology, comprising four principal modalities: chimeric antigen receptor (CAR)-T cells, T-cell receptor (TCR)-engineered T cells, tumor-infiltrating lymphocytes (TILs), and natural killer (NK) cells, including their CAR-engineered variants [32,33]. CAR-T cells, which employ synthetic receptors to effect MHC-independent cytotoxicity, have received multiple FDA approvals for hematological malignancies but face substantial barriers in solid tumors, including the immunosuppressive TME and antigen heterogeneity [34]. TIL therapy, now FDA-approved for advanced melanoma, offers a polyclonal, tumor-resident repertoire and has demonstrated objective responses in several solid tumor indications [35]. TCR-T cells extend targetable antigens to intracellular proteins and have recently achieved regulatory approval for synovial sarcoma, highlighting their potential in tumors with low surface-antigen density [36]. NK cells, distinguished by their intrinsic capacity for antibody-dependent cellular cytotoxicity and a favorable safety profile, are under active investigation in both native and CAR-engineered configurations [37]. A defining advantage of cellular immunotherapy is its capacity for potent, antigen-specific tumor elimination and the potential for durable immune memory [38,39].

Among these platforms, TIL therapy has shown notable promise in cervical cancer: a phase II trial reported objective responses in 5 of 18 patients (28%), including two complete responses ongoing beyond 53 months, with clinical benefit strongly correlated with HPV-specific T-cell reactivity [40]. Several CAR-T approaches, including those targeting PSMA, mesothelin, and HPV16 E6 via TCR-mimic nanobodies, are undergoing early-phase clinical or preclinical evaluation in cervical cancer, and a TCR-T trial targeting HPV16 E7 is also in clinical development [41]. Collectively, these advances underscore that cervical cancer, driven by persistent HPV oncoprotein expression, represents an especially compelling target for antigen-directed cellular immunotherapy.

The purpose of this review is to provide an overview of cellular immunotherapeutic strategies for cervical cancer, contextualized within the unique immunological barriers imposed by HPV-driven oncogenesis. We first delineate the immunosuppressive architecture of the cervical TME, with particular emphasis on mechanisms of HPV-mediated immune evasion, including MHC class I downregulation, induction of regulatory T cell and myeloid-derived suppressor cell infiltration, and chronic T cell exhaustion, that collectively undermine endogenous antitumor immunity and confer resistance to conventional immunotherapy. We next survey the preclinical landscape of adoptive cell therapy platforms in HPV-associated disease models, examining the biological rationale and *in vivo* efficacy of CAR-T cells, TCR-engineered T cells, TILs, and NK cell-based approaches. The translational landscape of these cell therapy modalities is then critically appraised through a detailed analysis of completed and ongoing clinical trials, highlighting both promising response signals and persistent obstacles to durable disease control. Finally, we delineate emerging avenues for therapeutic optimization, including next-generation engineering strategies to circumvent antigen escape, combinatorial regimens to remodel the TME, and novel targets to potentiate effector function. We conclude with a forward-looking perspective on the integration of cellular therapy into the cervical cancer treatment.

## 2 The Molecular and Cellular Microenvironment of the Cervical Cancer

**Figure 1:**
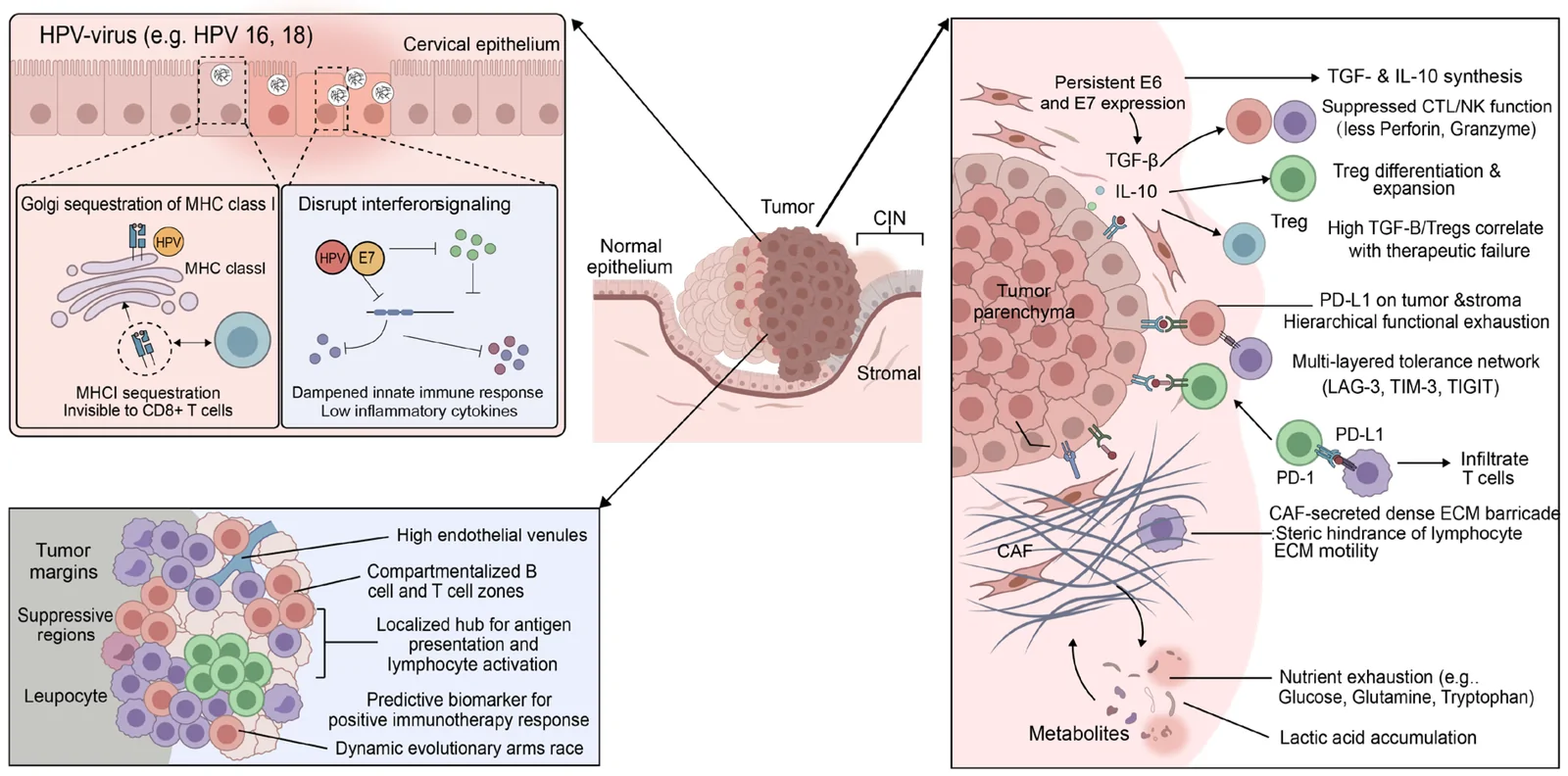
**Overview of the cervical cancer microenvironment.** Persistent expression of HPV oncoproteins, primarily E6 and E7 in established tumors, with E5 playing a more prominent role during early infection and viral persistence, induces transforming growth factor-beta (TGF-β) and interleukin (IL)-10, disrupts interferon signaling, and, in the case of E5, triggers Golgi sequestration of MHC class I molecules. E6 and E7 further contribute to immune evasion by downregulating antigen processing machinery and upregulating immune checkpoints. These changes dampen innate immunity, render tumor cells less visible to Cluster of Differentiation (CD)8^+^ T cells, and lower inflammatory cytokine levels. The figure depicts the invasive carcinoma setting, where E6/E7-driven immunosuppression predominates, though E5-related mechanisms may persist in a subset of tumors. The tumor stroma features high endothelial venules and compartmentalized B and T cell zones at cancer-associated fibroblast (CAF)-rich margins, creating local hubs for antigen presentation and lymphocyte activation that coexist with suppressive regions.

While the implementation of prophylactic vaccination and organized screening programs has led to a significant decline in incidence, the management of advanced, recurrent, and metastatic disease continues to pose substantial clinical challenges. A particularly pressing issue is the limited and variable response rates to emerging immunotherapies, such as immune checkpoint inhibitors. This therapeutic bottleneck is increasingly attributed not merely to the intrinsic properties of the malignant epithelial cells but to the complex, dynamically evolving TME. The cervical cancer TME constitutes a sophisticated ecosystem orchestrated by viral oncoproteins, metabolically reprogrammed tumor cells, heterogeneous stromal components, and a dysregulated immune network. A comprehensive dissection of the molecular and cellular architecture of this microenvironment, and the mechanisms underpinning its profoundly immunosuppressive nature, is therefore paramount for the rational design of novel therapeutic strategies and the improvement of patient outcomes (Fig. 1).

### 2.1 HPV Immune Evasion: Beyond Oncoproteins to Stealth Tactics

The high-risk HPV-encoded oncoproteins E5, E6, and E7 collectively orchestrate immune evasion, though their relative contributions differ across the viral life cycle and disease progression. During the initial, productive phase of HPV infection in the upper epithelial layers, the E5 oncoprotein plays a dominant role in facilitating viral persistence by enabling infected keratinocytes to evade innate and adaptive immune detection. As infection progresses to high-grade cervical intraepithelial neoplasia (CIN) and invasive carcinoma, E6 and E7 become the principal drivers of both cellular transformation and sustained immune suppression [42,43]. Their canonical roles involve E6-mediated degradation of the p53 tumor suppressor via the ubiquitin-proteasome pathway, compromising DNA damage repair and apoptosis, and E7-mediated binding and inactivation of the retinoblastoma protein (pRb), leading to unscheduled E2F-dependent cell cycle progression [44,45]. However, contemporary research reveals a far more extensive repertoire of immunomodulatory functions [46]. These oncoproteins enact a systematic and multi-layered reprogramming of the local immune landscape to establish an “immune-privileged” niche conducive to viral persistence and tumor progression [47].

The narrative began its significant expansion with the realization that for a virus to cause cancer, it must not only drive proliferation but also persist for decades, evading immune elimination [48]. This introduced the concept of viral immune evasion, where E6 and E7 were recast not just as intracellular transformers but as systemic regulators of the host immune landscape [49]. Early work identified mechanisms such as the downregulation of Major Histocompatibility Complex (MHC) class I molecules on the cell surface, impairing antigen presentation to CD8^+^ T cells. A key mechanism is mediated by the E5 oncoprotein, which binds to the MHC class I heavy chain and retains it in the Golgi apparatus, preventing its transport to the cell surface. This Golgi sequestration pathway is primarily attributed to E5, whereas E6 and E7 can also reduce surface MHC class I expression through distinct mechanisms, including transcriptional downregulation of MHC class I genes and impairment of the antigen processing machinery (e.g., TAP1/2), particularly in the context of established malignancies [50]. In the early, persistent phase of infection, E6 inhibits interferon regulatory factor-3 (IRF-3) activation, blunting the type I interferon response, a first line of antiviral defense, thereby allowing the virus to establish a long-term infection without eliciting sterile immunity [51]. E7 interferes with the expression of toll-like receptor 9 (TLR9), a sensor for viral DNA, further dampening innate recognition [52]. During progression to high-grade dysplasia and invasive cervical cancer, E6 and E7 continue to exert immunosuppressive effects while also driving genomic instability and uncontrolled proliferation. In this late stage, additional mechanisms emerge, including the upregulation of immune checkpoint ligands (e.g., PD-L1), recruitment of regulatory T cells and myeloid-derived suppressor cells, and secretion of immunosuppressive cytokines such as IL-10 and TGF-β [53]. These discoveries collectively frame the HPV oncoproteins as active, stage-dependent suppressors of innate and adaptive immune recognition.

In the setting of established invasive cervical cancer, a more complex immunosuppressive network emerges. Recently, Prins et al. elucidated a novel KLF2/IL-23 immunosuppressive axis directly driven by HPV16 E6/E7. This work demonstrated that tumor cells expressing these oncoproteins upregulate the expression of the transcription factor KLF2 in TAMs. KLF2, in turn, directly binds to and activates an enhancer of the IL-23 Agene, prompting macrophages to secrete copious amounts of interleukin (IL)-23. Contrary to its pro-inflammatory role in certain contexts, this IL-23 potently suppressed the proliferation, cytokine production, and cytotoxic function of HPV-specific CD8^+^ T cells. *In vivo*, neutralization of IL-23 reversed this suppression, increased intratumoral cytotoxic T lymphocyte infiltration, and delayed tumor growth, highlighting the therapeutic potential of targeting this pathway [54,55].

Beyond direct manipulation of myeloid cells, HPV has evolved sophisticated “stealth” mechanisms to evade early immune detection [56]. Viral DNA is trafficked within vesicles, avoiding intracellular pattern recognition receptors [57]. The low-level expression of early viral genes minimizes immunogenic signals, and HPV infection typically does not induce overt cytopathic effects or a robust inflammatory response, allowing the “invader” to go initially unnoticed [58]. Furthermore, the virus downregulates innate immune sensors in keratinocytes, suppresses type I interferon responses, impairs the expression of human leukocyte antigen molecules essential for antigen presentation, and recruits immunosuppressive cell populations like regulatory T cells (Tregs) [59,60]. This multi-pronged strategy effectively dismantles the host’s antiviral and antitumor defenses from the outset.

In summary, the immune-evasive functions of HPV oncoproteins are not static but evolve with disease progression. During early infection and viral persistence, E5-mediated Golgi retention of MHC class I, together with E6- and E7-mediated suppression of interferon and TLR9 signaling, allows the virus to establish a chronic infection without provoking a robust immune response. In premalignant lesions (CIN2/3), E6 and E7 begin to drive dysplasia while continuing to subvert antigen presentation and innate immunity. Finally, in invasive cervical cancer, E6 and E7 orchestrate a multilayered immunosuppressive tumor microenvironment characterized by checkpoint upregulation, recruitment of suppressive immune cell populations, and metabolic reprogramming. This temporal framework is essential for understanding why cellular immunotherapies—particularly those targeting E6/E7, may be most effective in the context of established tumors, whereas interventions aimed at reversing E5-mediated immune evasion might be more relevant for preventing progression from persistent infection to dysplasia.

### 2.2 The Metabolic and Stromal Fortress

The non-epithelial components of tumors were viewed as passive bystanders or merely structural scaffolds [61]. The stroma was the “soil” to the cancer cell’s “seed,” but its active role in pathogenesis was underappreciated [61]. Similarly, tumor metabolism, while recognized as altered since Warburg’s observations in the 1920s, was often considered a cell-autonomous adaptation for biomass production [62]. In cervical cancer, these two concepts have converged, revealing an active, tumor-promoting fortress built by metabolic cross-talk and stromal reprogramming.

The metabolic peculiarity of cancer cells, aerobic glycolysis or the Warburg effect, is a hallmark of cervical carcinoma. This shift from oxidative phosphorylation to lactate production, even in oxygen sufficiency, provides rapid ATP and critical biosynthetic precursors (nucleotides, amino acids, lipids) [63]. However, its impact extends beyond the cancer cell’s boundary [64]. The export of lactate into the extracellular space via monocarboxylate transporters acidifies the TME. This acidosis is not a benign byproduct; it is a potent immunosuppressive signal. It directly inhibits the cytolytic activity and cytokine production of T cells and NK cells while favoring the polarization of macrophages towards an M2-like, pro-tumorigenic phenotype and enhancing the suppressive function of Tregs [65]. Thus, a cell-intrinsic metabolic adaptation becomes a community-wide mechanism of immune exclusion [66,67]. It is important to note, however, that several immunosuppressive consequences of lactate accumulation, including direct inhibition of T cell and NK cell cytolytic activity and enhancement of regulatory T cell suppressive function, have been primarily established in other solid tumor models (e.g., melanoma, breast cancer) and extrapolated to cervical cancer. While the acidic pH of the cervical cancer TME has been documented, mechanistic studies specifically linking lactate to T cell exhaustion or Treg function in cervical carcinoma remain limited, representing an important area for future investigation.

The stromal compartment, far from being inert, is a dynamic and heterogeneous ecosystem actively educated by the tumor. Cancer-associated fibroblasts (CAFs), the most abundant stromal cell type, are now recognized as central players [68,69]. In cervical cancer specifically, multiple independent single-cell RNA-sequencing studies have identified distinct CAF subtypes, including inflammatory CAFs (iCAFs), myofibroblast-like CAFs (myCAFs), and antigen-presenting CAFs (apCAFs). They are not a uniform population but consist of multiple subsets with distinct origins and functions, as revealed by single-cell transcriptomics. Some subsets, like those expressing high levels of alpha-smooth muscle actin (α-SMA), contribute to desmoplasia, generating a dense, fibrotic extracellular matrix that acts as a physical barrier to immune cell infiltration [70]. Others are potent secretory hubs. For instance, inflammatory CAFs (iCAFs) secrete a cocktail of cytokines (e.g., IL-6, CXCL8) and growth factors that promote tumor cell proliferation, stemness, and angiogenesis. Recent spatial multi-omics studies have identified specific, organized niches within the TME [61]. One such niche features CD54-positive iCAFs in close spatial association with ITGAL-positive macrophages. This duo engages in a paracrine signaling loop involving CXCL8 and PDL1, creating a localized zone of potent T cell suppression [68]. Similarly, a distinct macrophage population characterized by high expression of osteopontin (SPP1) interacts with both cancer cells and exhausted T cells via SPP1-CD44 and fibronectin (FN1)-CD44 axes, further cementing immunosuppressive networks. Of note, while SPP1^+^ TAMs have been described across multiple solid tumor types, single-cell and spatial transcriptomic studies have demonstrated their significant enrichment specifically in cervical cancer compared to precancerous and normal cervical tissues, with high SPP1 expression correlating with advanced tumor stage and poor overall survival in cervical cancer patient cohorts [63]. These findings illustrate that the “stromal fortress” is not a random aggregation but a highly organized, spatially structured entity where specific CAF and immune cell subtypes co-localize to execute coordinated pro-tumor functions.

### 2.3 Tertiary Lymphoid Structures and Spatial Heterogeneity

The presence of lymphoid aggregates in solid tumors, later termed tertiary lymphoid structures (TLS), has been documented for years [71]. Initially, their detection was often simplistically correlated with a favorable prognosis across several cancer types, based on the premise that they represented sites of *de novo* anti-tumor immune activation, akin to lymph nodes [72]. This view cast TLS as a uniform biomarkers of immune engagement. Research in cervical cancer has been instrumental in challenging this simplistic notion and unveiling a profound layer of spatial and functional heterogeneity [73].

A real-world trial in specific aggressive subtypes, notably gastric-type endocervical adenocarcinoma (GAC). Contrary to expectation, the presence of TLS in GAC was associated with a worse prognosis and a more immunosuppressive overall TME signature. These TLS were found to be enriched not only in B cells but also in exhausted or dysfunctional T cell subsets and Tregs [74]. This pivotal finding established that TLS are not inherently immunogenic; their functional output, whether anti-tumor or pro-tumor, is context-dependent, influenced by the cytokine milieu, cellular composition, and likely the cancer genotype (e.g., HPV-related vs. HPV-independent) [75,76]. Subsequent research moved beyond mere presence/absence scoring to a more nuanced analysis of TLS architecture and location. A refined classification system emerged, considering both the maturity of the TLS (from early lymphocyte aggregates to fully formed structures with germinal centers) and their spatial distribution (intratumoral vs. peritumoral/invasive margin). This granularity revealed critical insights: mature, well-organized TLS located at the invasive margin of tumors are most strongly correlated with positive outcomes and response to immune checkpoint blockade. In contrast, immature or intra-tumoral TLS may lack functional organization or be influenced by different suppressive signals. This highlights that the quality and position of TLS are as important as their existence [77].

The latest layer of complexity is being uncovered through spatial transcriptomics and proteomics, allowing an unprecedented look inside these structures. These technologies have revealed that even within a single TLS, there can be significant functional zonation. For example, distinct plasma cell subsets secreting different antibody isotypes (e.g., IgA vs. IgG) may localize to different regions, potentially exerting opposing effects on tumor control [78]. Furthermore, the interaction between TLS and the surrounding TME is bidirectional. Immunosuppressive networks, such as those involving SPP1^+^ macrophages or specific CAF subsets, can infiltrate or influence the periphery of TLS, potentially “corrupting” their function [79]. This spatial interplay means that the TME cannot be understood as a uniform field but as a mosaic of distinct micro-niches, some immunologically “hot,” others “cold” or actively suppressive, with TLS acting as one key variable node whose role is determined by its specific location and cellular interactions [27,80].

As shown in Fig. 1, the schematic summarizes the complex, spatially organized immunosuppressive landscape of cervical cancer. Several features merit emphasis. First, persistent E6/E7 expression not only drives cell cycle dysregulation but also orchestrates a multi-layered immune evasion programme, including Golgi sequestration of MHC class I (primarily via E5), disruption of interferon signaling, and induction of TGF-β/IL-10, collectively rendering tumor cells poorly visible to CD8^+^ T cells. Second, the tumor stroma contains organized micro-niches: high endothelial venules facilitate lymphocyte entry, while CAFs at the tumor margin create compartmentalized B-cell and T-cell zones. These structures can serve as ectopic lymphoid hubs for antigen presentation, but they often coexist with suppressive regions enriched in regulatory T cells and myeloid-derived suppressor cells. Third, metabolites (e.g., lactate) further shape this ecosystem by polarizing macrophages toward an M2-like phenotype and inhibiting effector T-cell function. From a therapeutic perspective, Fig. 1 highlights why successful cellular immunotherapy must not only target HPV oncoproteins but also remodel the physical and metabolic barriers of the TME. The coexistence of “hot” and “cold” regions within the same tumor underscores the need for combination strategies that convert suppressive niches into permissive ones, and the spatial heterogeneity illustrated in the figure provides a rationale for using spatial biomarkers (e.g., TLS location and maturity) to predict response.

## 3 Tumor-Infiltrating Lymphocytes (TILs) in Cervical Cancer

### 3.1 HPV-Specific Reactivity in the TILs Repertoire

Cervical carcinogenesis is universally driven by persistent infection with high-risk HPV, predominantly HPV16 and HPV18, whose constitutively expressed E6 and E7 oncoproteins represent non-mutated, tumor-specific antigens restricted exclusively to malignant cells, thus forming the immunological core of the TIL repertoire in this malignancy [81,82]. Within the TME of HPV-driven cervical neoplasia, TILs comprise a heterogeneous mixture of CD8^+^ cytotoxic T lymphocytes, CD4^+^ helper T cells, and Treg cells, with the clonotypic architecture and functional potential of this compartment directly shaped by antigenic pressure from HPV E6 and E7 [61]. Deep profiling of TILs from syngeneic murine models of HPV+ cervical cancer and patient-derived tumor explants has revealed that the most extensively clonally expanded T cell populations within the TIL compartment are almost exclusively HPV-specific, bearing T cell receptors (TCRs) that recognize defined epitopes from the E6 and E7 oncoproteins [83]. These HPV-reactive T cells display a spectrum of functional states in preclinical systems: while a significant subset exhibits canonical hallmarks of T cell exhaustion, including co-expression of inhibitory receptors such as PD-1, TIM-3 and LAG-3, they retain intrinsic antigen reactivity that can be robustly reinvigorated upon *ex vivo* antigenic stimulation, as validated in tumor slice culture models and immune-competent mouse models of HPV+ cervical cancer [84,85]. Critically, preclinical adoptive transfer studies have confirmed that the *in vivo* anti-tumor efficacy of TILs is wholly dependent on the presence of these HPV-specific clones, with non-HPV-reactive bystander T cells showing negligible capacity to mediate tumor regression, establishing HPV reactivity as the foundational determinant of the therapeutic utility of cervical cancer TILs [86].

### 3.2 Ex Vivo Expansion Strategies

*Ex vivo* expansion strategies for TILs have evolved considerably through preclinical optimization, addressing the challenge of generating clinically relevant cell numbers from limited tumor specimens [87]. The classical expansion protocol employs a two-step process: an initial rapid pre-expansion phase, followed by an intensive rapid expansion phase utilizing high concentrations of IL-2 to drive T-cell proliferation [88]. This foundational approach has been refined through systematic investigation of cytokine combinations, with IL-7, IL-15, and IL-21 emerging as valuable supplements or alternatives to IL-2 that can influence T-cell differentiation, persistence, and functional properties. The development of “minimally cultured” TIL approaches represents another significant advancement, wherein all harvested T cells undergo rapid expansion without prior selection based on anti-tumor activity [89]. This strategy reduces production time and costs while preserving the natural heterogeneity of the TIL population, potentially offering broader antigen recognition capacity that could counteract tumor immune evasion through antigen loss. Preclinical models have also explored artificial antigen presentation during expansion to enhance the proportion of tumor-reactive clones, with continuous exposure to HPV antigens or neoantigens promoting the selective expansion of antigen-specific T cells [90]. The stochastic nature of T-cell clone expansion during culture presents a challenge, as inter-clonal competition can unpredictably alter the frequency of tumor-specific clones in the final product [83]. To address this, researchers have investigated methods to protect antigen specificity during expansion, including optimized culture conditions that maintain functional T-cell properties. The emergence of patient-derived organoid (PDO) systems has provided a transformative preclinical platform for studying TIL expansion and function, with biobanks of cervical cancer organoids accurately recapitulating parental tumor characteristics and enabling co-culture experiments with autologous TILs [91]. These three-dimensional models capture tumor heterogeneity and microenvironmental interactions more faithfully than traditional two-dimensional cultures, offering improved predictive value for therapeutic efficacy.

### 3.3 Determinants of Response and Resistance

Determinants of TIL response and resistance in cervical cancer constitute a complex interplay of cellular composition, functional states, and microenvironmental factors that preclinical studies have begun to unravel. The density and spatial distribution of TIL subsets within tumors emerge as critical variables, with higher overall TIL infiltration consistently associated with a more favorable prognosis across multiple preclinical models [92]. Specifically, CD8^+^ T-cell density at the invasive margin and within tumor stroma demonstrates predictive value for treatment outcomes, independent of PD-L1 expression status [93]. Functional polarization of TIL subsets further influences therapeutic potential, with responders typically exhibiting balanced Th1, Th2, Th17, and Treg profiles, while non-responders show predominance of exhausted CD8^+^ T cells and alternatively activated macrophages [93]. The immunosuppressive TME presents multiple barriers to TIL efficacy, including elevated expression of checkpoint molecules such as PD-1 and PD-L1 on TILs from non-responding tumors. Preclinical investigations have identified elevated levels of regulatory T cells with activated phenotypes in treatment-resistant settings, suggesting that Treg-mediated suppression represents a significant mechanism of immunotherapy resistance. Systemic immune mediators also contribute to response determination, with distinct cytokine profiles distinguishing responders from non-responders. Non-responding tumors exhibit higher levels of soluble mediators, including FGF-basic, IL-7, IL-8, IL-12p40, IL-15, and TNF-α, which correlate with specific TIL immune markers and may reflect broader immune dysregulation [83]. Although IL-7 and IL-15 are typically associated with T cell survival and proliferation, their presence in non-responding tumors could result from chronic inflammatory feedback, paradoxical induction of exhaustion markers, or production by non-T cells within the immunosuppressive microenvironment, warranting further mechanistic investigation. The functional capacity of TILs themselves represents another determinant, with telomere length, expression of costimulatory molecules CD27 and CD28, and differentiation status influencing proliferative potential and persistence. Preclinical models have further illuminated physical barriers to TIL efficacy, noting that less than 2% of transferred T cells typically infiltrate solid tumors, prompting investigation of strategies to enhance trafficking through modulation of chemokine receptors, adhesion molecules, and vascular normalization approaches [86,94]. These multifaceted determinants collectively shape the therapeutic potential of TILs in cervical cancer, highlighting the need for integrated assessment approaches in preclinical development.

## 4 Precision Targeting Based on TCR-Engineered T Therapies

### 4.1 Harnessing Viral Non-Self Tumor Antigens

T cell receptor (TCR)-engineered T cell therapy represents a paradigm shift in precision immunotherapy for cervical cancer, offering the potential to redirect the immune system with exquisite specificity against tumor cells [95,96]. This approach involves the genetic modification of patient-derived T cells to express TCRs that recognize specific peptide antigens presented by MHC molecules on the surface of cancer cells. The unique virology of cervical cancer, where over 90% of cases are driven by persistent infection with high-risk HPV types, provides an ideal framework for this strategy [97]. The constitutive expression of the viral oncoproteins E6 and E7 in transformed cells establishes them as perfect “non-self” tumor antigens, absent in healthy tissues, thereby minimizing the risk of on-target, off-tumor toxicity that plagues other targeted therapies [97]. Preclinical development has focused on isolating and engineering high-affinity TCRs against these viral antigens, optimizing expansion protocols, and understanding the biological determinants of efficacy and resistance within the complex TME [98].

The foundation of TCR-engineered therapy lies in harnessing the immunogenic potential of viral non-self tumor antigens, primarily the HPV-16 and HPV-18 E6 and E7 oncoproteins. These proteins are not only essential for malignant transformation and maintenance but are also highly conserved, making them stable targets less susceptible to immune editing through antigen loss [99]. Preclinical studies demonstrate the efficacy of genetically engineered T cells against cervical cancer through distinct strategies. Dall et al. showed that murine cytotoxic T lymphocytes retrovirally transduced with a chimeric receptor targeting the CD44v7/8 splice variant specifically lysed antigen-positive tumor cells and inhibited tumor growth in a xenograft model [100]. Scholten et al. successfully isolated TCR genes from HPV16E7-specific clones and transferred them into peripheral blood CD8^+^ T cells, generating transgenic T cells that recognized endogenously processed HPV16E7 epitopes, confirming TCR gene transfer as a viable therapeutic approach [101]. Complementing these experimental findings, Cho et al. developed a mathematical model indicating that successful tumor elimination by E7-targeting TCR T cells depends on a critical dose window relative to initial tumor size, and that combination therapy with IL-2 can improve outcomes in a cell line-specific manner [102].

A critical preclinical advance has been the successful isolation of TCR genes from HPV16E7-specific T cell clones derived from patients, demonstrating the feasibility of genetically engineering both CD8^+^ and CD4^+^ T cells to express these receptors. Engineered T cells expressing these TCRs have shown potent, antigen-specific recognition and cytotoxic activity against HPV-positive tumor cell lines *in vitro*. *In vivo* proof-of-concept has been robustly established in murine models, where adoptive transfer of E7-specific TCR-engineered T cells mediated significant regression of established HPV-16+ cervical cancer tumors [99]. Beyond the common HPV-16 genotype, strategies are expanding to cover other high-risk types. A notable example is the TCR (10F04), cloned from a long-term surviving patient, which is HLA-DRA/DRB1*09:01-restricted and specific for an HPV18 E7 epitope [103]. This TCR demonstrated robust antitumor activity in preclinical models, effectively redirecting both CD4^+^ and CD8^+^ T cells to specifically recognize and kill HPV18-positive tumor cells, highlighting the potential of broadening therapeutic coverage across multiple HPV genotypes [103]. The exploration extends beyond αβ T cells, with preclinical studies utilizing patient-derived cervical cancer organoids to evaluate the cytotoxicity of γδ T cells against HPV-transformed cells, revealing differential susceptibility and implicating pathways involving DNA damage response and ligands like BTN3A1 [104].

### 4.2 HLA Restriction and Mechanisms of Escape

A fundamental layer of complexity and a critical determinant of therapeutic efficacy is HLA restriction, which simultaneously defines precision and presents a major challenge for patient eligibility and tumor immune escape. TCRs recognize short peptide fragments presented by specific HLA alleles, creating a personalized therapeutic landscape. Preclinical work has successfully generated HPV-specific CD4^+^ T helper cells via TCR gene transfer using both MHC class I- and class II-restricted TCRs, demonstrating recognition of endogenously processed antigens presented by HLA-DP1 and HLA-A2, respectively [105]. This is crucial because effective and durable anti-tumor immunity requires coordinated help from CD4^+^ T cells. However, this allele-specific recognition inherently limits treatment to patients expressing the matching HLA type. To address this limitation, innovative “off-the-shelf” approaches are being explored preclinically. One strategy involves using induced pluripotent stem cell (iPSC)-derived, HPV-specific cytotoxic T lymphocytes that have been gene-edited to express specific HLA alleles (e.g., HLA-A24) while knocking out others to evade host rejection, creating a “limited off-the-shelf” product for patients with that HLA type [106]. This approach aims to overcome the logistical and manufacturing hurdles of patient-specific therapies.

Tumors employ multiple mechanisms to escape TCR-engineered T cell attack, many revolving around the antigen presentation machinery. Downregulation or complete loss of the restricting HLA allele is a primary escape route, rendering tumor cells invisible to the engineered T cells regardless of antigen expression [106]. Additionally, impaired antigen processing, such as mutations in the proteasome or transporter associated with antigen processing proteins, can prevent the viral oncoprotein-derived peptides from being loaded onto HLA molecules. The TME further contributes to resistance through immunosuppressive factors like regulatory T cells, MDSCs, and expression of checkpoint ligands like PD-L1, which can induce exhaustion in the infused TCR-engineered T cells [107]. Preclinical models, particularly patient-derived organoids, have become invaluable for studying these dynamics. Small biobanks of cervical pre-tumoroids and tumoroids that faithfully retain genomic characteristics and the causative HPV genome enable co-culture experiments with immune cells [107]. These systems allow researchers to model tumor-immune interactions, screen therapeutic peptides, and observe differential responses to immunized peripheral blood mononuclear cells, providing a more physiologically relevant platform to identify and overcome mechanisms of resistance.

## 5 Overcoming the Solid Tumor Barrier Using CAR-T Cells

### 5.1 Target Antigen Selection in Cervical Cancer

Overcoming the formidable barrier of solid tumors, including cervical cancer, requires sophisticated engineering of CAR T cells, focusing on precise antigen selection, enhanced cellular potency, and active remodeling of the immunosuppressive TME. Target antigen selection is paramount to ensure efficacy while minimizing on-target, off-tumor toxicity. Preclinical screening in cervical squamous cell carcinoma has identified Mucin 1 (MUC1) and Mesothelin (MSLN) as promising tumor-associated antigens due to their high expression in tumor tissues compared to normal cervical epithelium [84]. Beyond these, other antigens like MUC16 (CA125) and EGFR are under investigation for their roles in tumorigenesis [108]. A critical advancement is targeting specific oncogenic isoforms, such as the growth factor receptor form MUC1, with CARs designed to avoid recognition of full-length MUC1 on healthy cells, thereby improving specificity [109,110]. The selection often extends to viral neoantigens like the HPV E6/E7 oncoproteins, offering truly tumor-specific targets, though their presentation can be limited by HLA restriction.

Recent preclinical studies have extensively explored CAR T-cell therapy for cervical cancer, evaluating a diverse array of target antigens to overcome the challenges of solid tumors. Research has demonstrated potent *in vitro* and *in vivo* antitumor activity of CAR-T cells targeting MSLN [111], the immune checkpoint molecule CD155 [112], placental alkaline phosphatase (PLAP) [113], the stress ligand NKG2D [114], and epidermal growth factor receptor (EGFR) [115]. Innovative strategies to enhance specificity and potency include designing TCR-mimic nanobodies against the HPV16 E6 oncoprotein presented by HLA-A*02:01 [41] and engineering NK-92 cells to co-express an HPV16 E7-specific TCR with a costimulatory trophoblast cell surface protein 2 (TROP2)-targeting CAR, which synergistically enhanced cytotoxicity [116]. However, a critical study highlights a major barrier in the hypoxic TME, showing that the HIF-1α inhibitor PX-478 can unexpectedly impair the antigen-specific cytotoxic function of MESN-CAR T cells and promote exhaustion [117], underscoring the complex interplay between combination therapies and CAR-T cell efficacy. Collectively, these studies validate multiple antigen targets and engineering strategies while emphasizing that the immunosuppressive TME remains a pivotal determinant of therapeutic success.

### 5.2 Weaponizing the Cellular Graft: Fourth and Fifth-Generation Armored CARs

To weaponize the cellular graft against the hostile TME, fourth and fifth-generation “armored” CARs have been developed. Fourth-generation CARs (TRUCKs) incorporate an inducible transgene system, typically driven by an NFAT-responsive promoter, to secrete immunomodulatory cytokines such as IL-12 upon antigen engagement [118]. While this localized delivery elegantly bypasses systemic toxicity, the IL-12 payload is a double-edged sword: it potently recruits and activates innate immune cells, but sustained or excessive IL-12 signaling can drive T-cell exhaustion and even induce fatal inflammatory toxicities in preclinical models, an underappreciated trade-off that warrants cautious titration. Fifth-generation CARs add a truncated cytoplasmic domain from cytokine receptors (e.g., IL-2Rβ chain) to integrate JAK-STAT signals alongside CD3ζ and CD28 co-stimulation [119,120]. In theory, this tripartite signal should enhance memory formation and persistence; however, the actual benefit over optimized second-generation or TRUCK constructs remains unproven in head-to-head comparisons, particularly in solid tumors where antigen heterogeneity and metabolic stress dominate. Notably, cervical cancer presents a unique opportunity and challenge: the abundant HPV-antigen landscape enables tumor recognition, yet the fibrotic, hypoxic TME rich in cancer-associated fibroblasts (CAFs) and regulatory T cells (Tregs) imposes physical and metabolic barriers that even armored CARs struggle to overcome. Preclinical models targeting MSLN in cervical cancer have explored genetic knockdown of immunosuppressive receptors like A2aR and Tim3 to prevent exhaustion [121]. While promising, these strategies raise a critical question: does removing one inhibitory receptor merely shift the suppression to another compensatory pathway (e.g., Lag-3 or TIGIT)? The field may need to move beyond single-receptor editing toward multiplexed genetic or epigenetic reprogramming that rewires the entire exhaustion circuit. Overall, fourth and fifth-generation CARs represent logical engineering steps, but their clinical translation in cervical cancer will require rigorous benchmarking against simpler constructs and deeper integration with TME-modulating agents rather than assuming that added signaling complexity automatically yields superior outcomes.

## 6 The Allogeneic Frontier: Innate Effectors and CAR-NK Cell Therapies

### 6.1 Bypassing MHC Downregulation Via Innate Recognition

A fundamental advantage of NK cells in allogeneic cell therapy is their ability to recognize and eliminate tumor cells that have downregulated MHC-I molecules, a common immune evasion strategy in cancers like cervical carcinoma. NK cells employ “missing-self” recognition, where the absence of inhibitory MHC-I ligands on target cells releases the brake on NK cell activation, leading to cytotoxic killing [122]. This innate mechanism allows CAR-NK cells to target tumors that may escape T-cell-based therapies reliant on MHC-presented antigens, providing a critical layer of defense against antigen loss variants.

Preclinical studies demonstrate the promise of CAR-NK cell therapies for cervical cancer, utilizing diverse targeting strategies. One innovative approach engineers NK-92 cells to co-express a TCR against an HPV16 E6 epitope and a CAR against the tumor-associated antigen L1CAM, creating a synergistic “AND-gate” system that enhances both cytotoxicity and specificity upon dual antigen engagement [123]. Parallel research has validated third-generation CAR-NK cells targeting specific single antigens highly expressed in cervical tumors. These include anti-fibroblast activation protein (FAP) CAR-NK cells, which effectively lyse both cervical cancer cells and CAFs in 2D and 3D models, highlighting a strategy to remodel the TME [124], and anti-mesothelin CAR-NK cells, which show potent, antigen-specific cytotoxicity that can be further enhanced in combination with chemotherapy [125]. Collectively, these studies, employing both NK-92 cell lines and primary NK cells, confirm the potent and specific antitumor activity of CAR-NK cells against cervical cancer in preclinical models, supporting their development as a viable allogeneic therapeutic platform.

### 6.2 iPSC Derivation and Universal off-the-Shelf Platforms

iPSCs have emerged as a transformative source for generating standardized, scalable, and genetically uniform CAR-NK cells, enabling true “off-the-shelf” therapeutics. iPSCs can be efficiently differentiated into NK cell progenitors and mature effector cells, with genetic engineering (e.g., CAR insertion, cytokine armoring, checkpoint knockout) performed at the pluripotent stage to ensure batch-to-batch consistency [106,126,127]. This platform overcomes the limitations of donor-dependent sources (like peripheral or cord blood) and allows for the creation of homogeneous, pre-manufactured cell banks, significantly reducing production time and cost while facilitating immediate clinical availability [128].

## 7 Comparative Analysis of Adoptive Cell Therapy Platforms for Cervical Cancer

**Figure 2:**
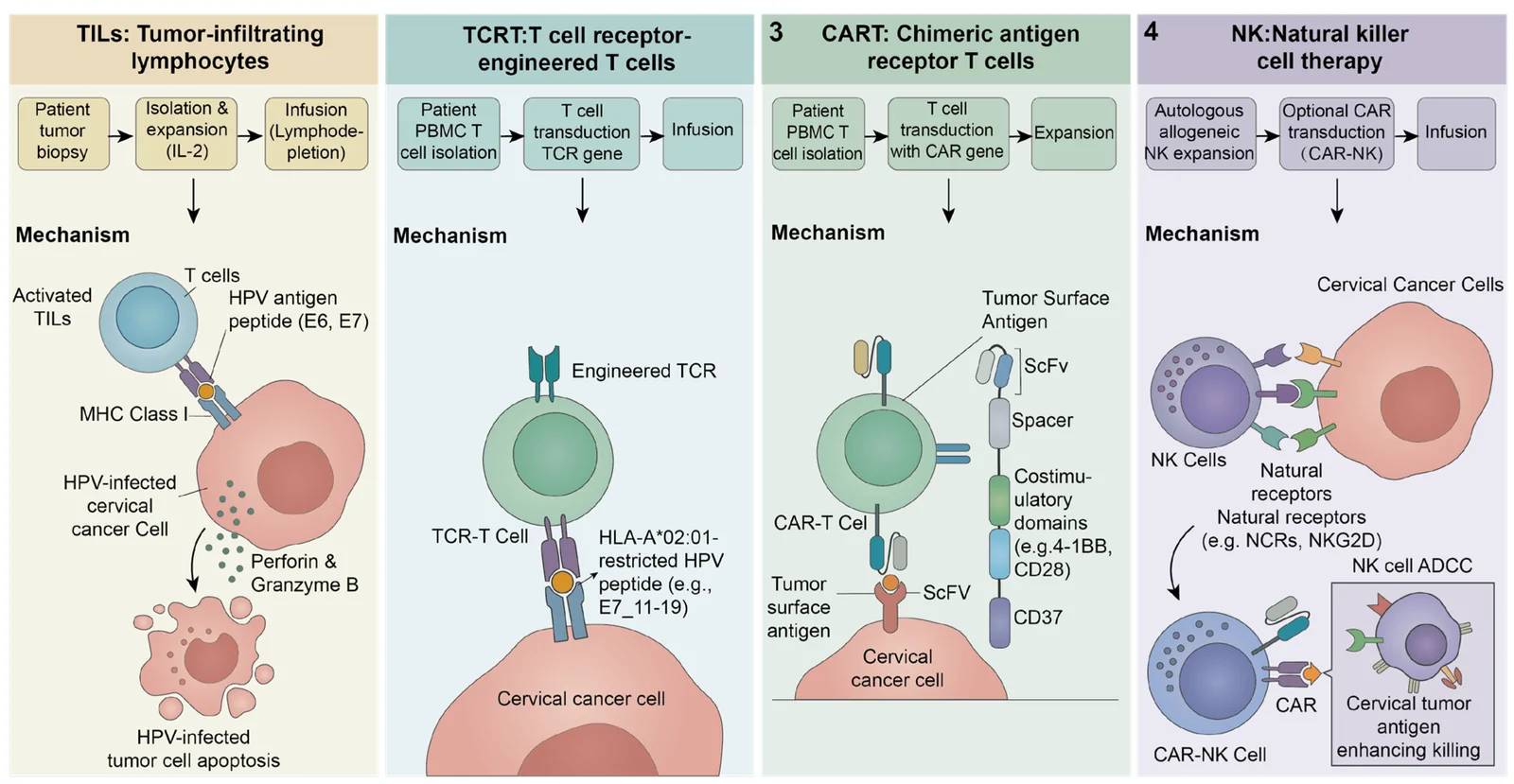
**Schematic overview of adoptive cell therapy strategies for cervical cancer and their mechanisms of action.** The figure compares four cellular immunotherapies for cervical cancer: TIL therapy (isolate, expand, reinfuse to kill HPV-peptide/MHC I targets via perforin/granzyme B); TCR-T therapy (engineer T cells with HPV-specific TCR to recognize peptide–MHC complexes); CAR-T therapy (engineer T cells with CAR containing scFv, costimulatory domains, and CD3ζ to kill surface-antigen-positive tumor cells); and NK cell therapy (expand autologous/allogeneic NK cells, optionally with CAR, to kill via natural receptors like NKG2D).

The landscape of adoptive cell therapy for cervical cancer features four distinct platforms, each with unique mechanisms and trade-offs. TILs utilize an autologous, polyclonal repertoire, offering broad antigen recognition and the potential for durable responses, albeit through a logistically complex, patient-specific process. TCR-T cells provide precise targeting of intracellular viral oncoproteins like HPV E6/E7, but their efficacy is restricted by specific HLA haplotypes. CAR-T cells enable potent, MHC-independent attack on surface antigens, yet their application in solid tumors like cervical cancer is hampered by the hostile TME and significant toxicities. In contrast, CAR-NK cells combine innate tumor recognition with engineered specificity, presenting a favorable safety profile and promising “off-the-shelf” potential, though their persistence *in vivo* requires further optimization. Fig. 2 contrasts the four principal adoptive cell therapy platforms for cervical cancer. TILs are harvested directly from the patient’s tumor, expanded *ex vivo* with IL-2, and reinfused after lymphodepletion. Because they are polyclonal and contain HPV-specific clones, they recognize multiple E6/E7-derived epitopes presented by MHC class I, leading to perforin/granzyme-mediated killing. This MHC-dependent mechanism explains why TILs are effective even when tumor cells downregulate individual MHC alleles, but also why complete MHC loss confers resistance. TCR-T cells are engineered from peripheral blood T cells to express a high-affinity TCR specific for an HPV peptide-MHC complex (e.g., HLA-A*02:01-restricted E7). Their MHC restriction provides precise intracellular targeting but limits patient eligibility to those carrying the relevant HLA allotype. CAR-T cells use a chimeric antigen receptor comprising an scFv against a tumor surface antigen (e.g., mesothelin, CD70), coupled with costimulatory domains (4-1BB, CD28) and CD3ζ. This MHC-independent recognition enables potent activation but risks on-target/off-tumor toxicity if the antigen is also expressed on healthy epithelia. CAR-NK cells combine innate recognition (via natural cytotoxicity receptors NKG2D, NCRs) with a CAR targeting a tumor antigen. The innate arm provides missing-self recognition, allowing elimination of MHC-low tumor variants, while the CAR enhances specificity. Their favorable safety profile (minimal CRS/ICANS) and potential for off-the-shelf allogeneic use are major advantages, though limited *in vivo* persistence remains a challenge. Together, these platforms represent a complementary arsenal, with the optimal choice depending on the antigenic landscape, patient genetics, and the need to overcome specific tumor escape mechanisms (Table 1, Fig. 2).

As the efficiency, TIL shave demonstrated durable complete responses (28% objective response rate, two complete responses ongoing beyond 53 months in a phase II trial) and are not HLA-restricted, but their autologous nature requires a 22-day manufacturing process and fresh tumor tissue, limiting scalability. TCR-T cells targeting HPV16 E7 have shown superior activity to E6-directed constructs and achieve potent intracellular viral antigen recognition; however, their HLA-restriction (e.g., HLA-A*02:01) narrows patient eligibility, and off-target cross-reactivity remains a safety concern. By contrast, CAR-T cells offer MHC-independent, high-affinity targeting of surface antigens and can be produced from peripheral blood, but their efficacy in cervical cancer has been constrained by antigen heterogeneity, poor tumor trafficking, and the immunosuppressive microenvironment. Moreover, on-target/off-tumor toxicity (e.g., against mesothelin or CD70 on normal epithelia) has proved dose-limiting, and no CAR-T product has yet achieved regulatory designation for cervical cancer. CAR-NK cells provide an off-the-shelf alternative with a favorable safety profile but currently show limited *in vivo* persistence. Thus, while CAR-T cells are conceptually powerful, their clinical feasibility and efficacy in cervical cancer lag behind TILs and TCR-T cells, underscoring the need for next-generation engineering (armored CARs, logic gates) and rational combinations to overcome solid-tumor barriers.

**Table 1:** Comparison of adoptive cell therapy platforms for cervical cancer.

Therapy	Mechanism	Key Advantage	Main Challenge	Clinical Stage	References
**TILs**	Autologous polyclonal T cells	Durable complete responses	Complex manufacturing	Phase I/II	[82,129]
**TCR-T**	HLA-restricted intracellular targeting	Targets viral oncoproteins (E6/E7)	HLA restriction	Phase I/II	[95,99]
**CAR-T**	MHC-independent surface antigen targeting	Potent, tunable activation	Solid tumor barriers & CRS	Phase I/II	[26,130]
**CAR-NK**	Innate cytotoxicity + CAR targeting	Favorable safety & “off-the-shelf” potential	Limited *in vivo* persistence	Preclinical	[131]

Abb.: TIL: Tumor-Infiltrating Lymphocyte, TCR: T Cell Receptor, CAR: Chimeric Antigen Receptor, NK: Natural Killer cell, HLA: Human Leukocyte Antigen, MHC: Major Histocompatibility Complex, CRS: Cytokine Release Syndrome.

## 8 The Clinical Trial Landscape of Cellular Therapy in Cervical Cancer

### 8.1 Current Clinical Trial Landscape

TIL therapy currently leads the clinical translation race in cervical cancer [86]. Because cervical cancer is a virally driven “hot” tumor, native T cells infiltrating the TME naturally possess a polyclonal capacity to recognize multiple HPV viral oncoproteins and tumor neoantigens [95]. Pioneering phase II trials conducted by the National Cancer Institute (NCI) demonstrated that a single infusion of HPV-targeted TILs could mediate durable, complete regressions in heavily pretreated patients with metastatic cervical cancer, with some patients achieving over a decade of continuous disease-free survival, effectively representing a clinical cure [40]. Building upon this foundational proof-of-concept, the investigational autologous TIL product LN-145 (lifileucel) has been extensively evaluated in the global, multicenter C-145-04/innovaTIL-04 phase II trial (NCT03108495). It is important to note that while lifileucel has received FDA accelerated approval for unresectable or metastatic melanoma, it remains an investigational therapy for cervical cancer, having been granted FDA Breakthrough Therapy Designation for this indication rather than full marketing authorization. Following promising objective response rates and high disease control rates in patients who failed standard systemic therapies, LN-145 received Breakthrough Therapy Designation from the US Food and Drug Administration (FDA), marking a watershed moment for cellular therapies in gynecologic oncology.

Concurrently, TCR-T cell therapy offers a more targeted, gene-engineered approach by equipping peripheral blood T cells with high-affinity receptors specific to intracellular antigens presented by major histocompatibility complex (MHC) molecules [129]. Given that the HPV E6 and E7 oncoproteins are constitutively expressed in cervical cancer cells to maintain the malignant phenotype but are absent in healthy tissues, they serve as ideal, non-self therapeutic targets [132]. Clinical trials have revealed a divergence in efficacy between these two targets; while phase I/II trials evaluating E6-targeted TCR-T cells (e.g., NCT02280811) reported limited objective responses, E7-targeted TCR-T cells (e.g., NCT02858310, NCT05686226) have demonstrated superior clinical and pre-clinical activity, including durable complete responses [133,134]. Furthermore, to expand applicability beyond virally driven antigens, novel TCR-T trials are exploring cancer-germline antigens such as KK-LC-1 (NCT05483491) in pan-solid tumor cohorts that include cervical cancer. In a single-center phase I clinical trial (NCT04443296), the results demonstrated that adjuvant autologous TIL therapy administered after concurrent chemoradiotherapy has a favorable safety profile, 74.1% feasibility for successful Good Manufacturing Practice (GMP)-compliant TIL manufacturing, and promising preliminary antitumor activity with a 75.0% complete response rate in treated patients with stage III-IV locally advanced cervical cancer, while translational investigations further revealed that transcriptomic characteristics of infused TIL products and baseline immune biomarkers in the TME and patient serum are correlated with clinical response, confirming that an inflamed “hot” immune microenvironment benefits the efficacy of this TIL-based adjuvant immunotherapy [135].

In contrast, CAR-T cell therapy, which relies on MHC-independent recognition of surface antigens, remains in the nascent stages of exploration for cervical cancer. The major bottleneck for CAR-T in solid tumors is the scarcity of highly specific surface antigens [130,136]. Current phase I and II clinical trials for cervical cancer are evaluating CAR constructs directed against MSLN (NCT01583686), the TROP2, CD22 (NCT04556669), CD70 (NCT05518253, NCT05468190, NCT05420545), and placental alkaline phosphatase (ALPP) (NCT04627740). Additionally, multi-target platforms (NCT03356795) assessing CAR-T cells specific to GD2, PSMA, MUC1, or MSLN simultaneously aim to counteract antigen heterogeneity and prevent immune escape. Lastly, innate immune cell therapies, particularly CAR-NK cells, are rapidly gaining traction due to their HLA-independent cytotoxicity and favorable safety profiles. Trials evaluating cord blood-derived, IL-15-armored TROP2-CAR-NK cells (NCT06066424), a cervical cancer-specific trial, represent the leading edge of transitioning from autologous, bespoke products to ‘off-the-shelf’ universal cellular therapeutics. A detailed breakdown of trial designs, including distinction between cervical cancer-specific studies and broader basket or HPV-associated malignancy trials, is provided in Table 2.

**Table 2:** Clinical trials of cellular immunotherapies for cervical cancer (completed, active, or recruiting as of May 2026, by clinicaltrial.gov).

NCT Number	Tumor Type	Treatment Method	Drug Name	Strategy	Phase	Status	Number of Patients
NCT03108495	Cervical cancer-specific	TIL	LN-145	Monotherapy/Combination with Pembrolizumab	Phase 2	Terminated	210
NCT01585428	HPV-associated malignancies	TIL	HPV-TILs	Autologous TILs targeting HPV E6/E7	Phase 2	Completed	29
NCT04443296	Cervical cancer-specific	TIL	TIL	Cisplatin concurrent chemoradiotherapy plus TIL	Phase 1	Unknown	10
NCT04674488	Cervical cancer-specific	TIL	Autologous TIL	TIL infusion following non-myeloablative lymphodepletion	Phase 1	Recruiting	15
NCT06630611	Pan-solid tumor	TIL	Autologous TIL	TIL therapy for refractory solid tumors	Phase 2	Recruiting	40
NCT02858310	HPV-associated malignancies	TCR-T	E7 TCR T Cells	Gene-engineered TCR targeting HPV-16 E7	Phase 1/Phase 2	Completed	224
NCT05686226	HPV-associated malignancies	TCR-T	E7 TCR T Cells	Gene-engineered TCR targeting HPV-16 E7	Phase 2	Recruiting	20
NCT05639972	HPV-associated malignancies	TCR-T	E7 TCR T Cells	Gene-engineered TCR targeting HPV-16 E7	Phase 1/Phase 2	Recruiting	15
NCT02280811	HPV-associated malignancies	TCR-T	E6 TCR T Cells	Gene-engineered TCR targeting HPV-16 E6	Phase 1/Phase 2	Completed	12
NCT03578406	HPV-associated malignancies	TCR-T	αPD1-TCR T Cells	TCR targeting HPV-16 E6 with PD-1 pathway blockade	Phase 1	Completed	9
NCT05483491	Pan-solid tumor	TCR-T	KK-LC-1 TCR-T	TCR targeting cancer-germline antigen KK-LC-1	Phase 1	Recruiting	30
NCT04556669	Pan-solid tumor	CAR-T	A PD-L1 armored anti-CD22 CAR-T/CAR-TILs	CD22 target with autonomous anti-PD-L1 secretion	Phase 1	Recruiting	30
NCT03356795	Cervical cancer-specific	CAR-T	CC-specific CAR-T	Multi-target approach (GD2, PSMA, MUC1, MSLN)	Phase 1/Phase 2	Recruiting	20
NCT05518253	Pan-solid tumor	CAR-T	CD70 CAR-T	Targeting CD70 surface antigen	Phase 1	Recruiting	30
NCT05468190	Pan-solid tumor	CAR-T	CD70 CAR-T	Targeting CD70 surface antigen	Phase 1	Recruiting	48
NCT05420545	Pan-solid tumor	CAR-T	CD70 CAR-T	Targeting CD70 surface antigen	Phase 1	Recruiting	36
NCT06010875	Pan-solid tumor	CAR-T	CD70 CAR-T	Targeting CD70 surface antigen	Phase 1	Recruiting	48
NCT04627740	Pan-solid tumor	CAR-T	Anti-ALPP CAR-T	Targeting Placental Alkaline Phosphatase (ALPP)	Phase 1/Phase 2	Completed	5
NCT01583686	Pan-solid tumor	CAR-T	Anti-Mesothelin CAR-T	Targeting Mesothelin surface antigen	Phase 1/Phase 2	Terminated	15
NCT06939270	Pan-solid tumor	CAR-T	CD73/AXL HypoSti.CAR-T	Dual-targeting CD73 and AXL to block immunosuppression	Phase 1/Phase 2	Not yet recruiting	30
NCT06066424	Pan-solid tumor	CAR-NK	TROP2-CAR-NK	Cord blood-derived allogeneic NK cells targeting TROP2	Phase 1	Recruiting	54
NCT06239220		NK Cell	PD-L1 t-haNK	Combination with IL-15 superagonist (N-803) and Cetuximab	Phase 2	Recruiting	25

Abb.: HPV: Human Papillomavirus; PD-L1: Programmed Death-Ligand 1; CD22: Cluster of Differentiation 22; GD2: Ganglioside GD2; PSMA: Prostate-Specific Membrane Antigen; MUC1: Mucin 1; MSLN: Mesothelin; ALPP: Alkaline Phosphatase, Placental type; AXL: AXL receptor tyrosine kinase; IL-15: Interleukin-15.

### 8.2 Combination Therapy Strategies

Despite the unprecedented potential of cellular therapies, the hostile and profoundly immunosuppressive TME of cervical cancer poses a formidable barrier to the persistence, infiltration, and effector function of adoptively transferred cells. Consequently, monotherapy approaches are increasingly being superseded by rational combination strategies designed to synergistically remodel the TME, mitigate T-cell exhaustion, and amplify the depth of the immunological response [84].

The most prominent combination strategy involves the concurrent administration of ACT and immune checkpoint inhibitors, primarily targeting the PD-1/PD-L1 axis [137]. Upon entering the solid tumor bed, TILs and CAR-T cells rapidly upregulate inhibitory receptors as a consequence of chronic antigen exposure and metabolic starvation, leading to a state of profound exhaustion. Administering PD-1 inhibitors alongside cellular products essentially “releases the brakes” on the engineered cells. This is exemplified by the innovaTIL-04 trial (NCT03108495, Arm 3), which demonstrated that combining LN-145 TILs with pembrolizumab in earlier lines of therapy resulted in heightened response rates. To avoid the systemic toxicities of co-administered monoclonal antibodies, next-generation “armored” CAR-T therapies (such as the anti-CD22 CAR-TILs evaluated in NCT04556669) are engineered to autonomously secrete anti-PD-L1 antibodies locally within the tumor bed, ensuring high localized concentrations while sparing peripheral tissues.

Furthermore, integrating cellular therapy with traditional modalities, such as chemoradiotherapy, is emerging as a potent combinatorial framework. Standard-of-care radiation and certain chemotherapeutic agents (e.g., cisplatin) can induce immunogenic cell death (ICD), a process characterized by the release of damage-associated molecular patterns (DAMPs) and pro-inflammatory cytokines [16]. This process effectively converts an immunologically “cold” TME into a “hot” one, facilitating robust chemokine-driven recruitment and tumor infiltration of subsequently infused T cells or NK cells [16]. For example, NCT01194609 explores the use of low-dose radiation as a preconditioning strategy to reverse the immune-compromised environment specifically for recurrent cervical cancer prior to adoptive immune cell transfer.

Targeted therapies and epigenetic modulators also present highly rational combination partners. Enhancers of Zeste Homolog 2 (EZH2) inhibitors and Bromodomain and Extra-Terminal motif (BET) inhibitors (e.g., PLX2853) are being actively investigated for their capacity to epigenetically upregulate the expression of target antigens (such as GD2 or mesothelin) on the surface of cervical cancer cells, thereby increasing the avidity and recognition capacity of CAR-T cells while simultaneously repressing pro-tumorigenic stroma signals [138]. Additionally, multi-modal regimens leveraging antibody-dependent cellular cytotoxicity are under clinical evaluation. The phase II trial NCT06239220 utilizes a sophisticated tri-combination: high-affinity PD-L1 targeted NK cells (t-haNK), the IL-15 superagonist N-803 to fuel NK cell proliferation, and the anti-EGFR monoclonal antibody cetuximab to orchestrate a coordinated innate and adaptive immune attack against HPV-driven malignancies.

### 8.3 Safety Considerations

While the therapeutic ceiling of cellular therapies is extraordinarily high, this potential is matched by a complex and potentially life-threatening toxicity profile that demands specialized clinical infrastructure for safe management [139]. The primary safety concerns intrinsic to ACT include cytokine release syndrome (CRS), immune effector cell-associated neurotoxicity syndrome (ICANS), on-target/off-tumor toxicities, and complications arising from the requisite preconditioning regimens [140].

Cytokine release syndrome is a systemic inflammatory response triggered by the explosive *in vivo* expansion and activation of adoptively transferred T cells upon encounter with their cognate antigen. This rapid activation precipitates a massive release of pro-inflammatory cytokines, prominently IL-6 and interferon-gamma, leading to fevers, hypotension, capillary leak syndrome, and, in severe cases, multiorgan failure [141]. Although severe (Grade 3 or higher) CRS and ICANS are markedly more prevalent in hematological CAR-T applications, early-phase solid tumor trials, including those for advanced cervical cancer, still report high frequencies of low-grade CRS that necessitate vigilant monitoring and prompt intervention with tocilizumab (an IL-6 receptor antagonist) or corticosteroids [142].

A unique and profound safety challenge in treating cervical cancer with CAR-T and TCR-T therapies is on-target, off-tumour toxicity [143]. Unlike the CD19 antigen in B-cell lymphomas, where complete B-cell aplasia is a clinically manageable side effect, solid tumor-associated antigens such as mesothelin, CD70, and human epidermal growth factor receptor 2 (HER2) are frequently expressed at basal levels on vital healthy epithelia. High-affinity CAR-T cells cannot easily discriminate between the overexpression of these antigens on a cervical tumour and their physiological expression on the pleura or pericardium, which has historically led to fatal pulmonary and cardiac toxicities in early solid tumour trials [144]. To mitigate these risks, bioengineers are actively developing sophisticated synthetic biology ‘safety switches’, such as inducible caspase-9 (iCasp9) suicide genes, and logic-gated CAR networks (for example, NOT-gated systems designed to spare HLA-A*02-positive healthy tissues) to ensure tight spatial and temporal control over engineered cells [145].

Beyond the cellular products themselves, the preparative regimens required for successful ACT carry substantial morbidity [146]. Standard lymphodepletion protocols, typically comprising high-dose fludarabine and cyclophosphamide, are mandatory to eradicate host Tregs and endogenous ‘cytokine sinks’, thereby creating a favorable immunological niche for the engraftment of infused therapeutic cells [147,148]. However, this regimen universally induces profound and prolonged bone marrow suppression, rendering patients highly susceptible to opportunistic infections and bleeding diatheses [149]. Furthermore, the traditional TIL therapy protocol mandates post-infusion administration of high-dose systemic IL-2 (aldesleukin) to sustain T-cell expansion, a treatment notorious for causing severe hypotension, vascular leak, and renal impairment [88]. Next-generation strategies using CAR-NK cells engineered to co-express tethered IL-15 (e.g., NCT06066424) aim to provide autocrine survival signals, thereby eliminating the need for toxic systemic cytokine administration.

TIL therapy exhibits a distinct safety profile dominated by lymphodepletion-related cytopenias and high-dose IL-2-driven capillary leak syndrome, with severe CRS in <10% of patients and virtually no ICANS. TCR-engineered T cells carry an added risk of on-target/off-tumor cross-reactivity against peptide-MHC complexes on healthy tissues, though this is mitigated for HPV-restricted antigens, and grade ≥ 3 CRS occurs in 15–25% of recipients without routine post-infusion IL-2. CAR-T cells show the most fulminant toxicity, with CRS in 70–90% of patients, frequent ICANS, and dose-limiting on-target/off-tumor attack driven by basal antigen expression on normal epithelia, which precludes IL-2 support. CAR-NK cells afford a markedly safer profile, with minimal CRS (<5% grade ≥ 3) and rare ICANS owing to a distinct cytokine output, limited *in vivo* expansion, and inhibitory-receptor-mediated self-regulation. Lymphodepletion is mandatory for TIL, TCR-T, and CAR-T engraftment but is often reduced or omitted for CAR-NK therapies; post-infusion IL-2 is required only for TILs, whereas next-generation CAR-NK constructs incorporate membrane-bound IL-15 to obviate systemic cytokine support. Emerging synthetic safety switches, inducible caspase-9, logic-gated AND/NOT CARs, and small-molecule-controlled on/off switches, are being developed to overcome the on-target/off-tumor toxicity that remains a central barrier for CAR-T application in cervical cancer.

### 8.4 Manufacturing and Logistical Challenges

Translating cellular therapies from highly controlled academic laboratories to global, standardized clinical care for cervical cancer presents staggering manufacturing and logistical hurdles [150]. The fundamental challenge lies in the bespoke, autologous nature of current FDA-approved and late-stage experimental products [151].

The most critical clinical limitation is “vein-to-vein time”, the interval from the initial surgical excision of the tumor (for TILs) or leukapheresis of peripheral blood (for CAR-T/TCR-T) to the final infusion of the manufactured product back into the patient [152]. For therapies like LN-145, the central manufacturing process dictates an intricate 22-day expansion protocol. Including shipping, quality control, release testing, and patient preconditioning, the overall turnaround time can span four to six weeks. For patients with rapidly progressing metastatic cervical cancer, this delay results in substantial attrition rates, as many patients either experience catastrophic clinical deterioration or succumb to their disease before the therapy can be administered.

Logistically, the supply chain for autologous cell therapies requires an unyielding cold chain and a flawless “chain of identity” and “chain of custody” tracking system [153]. Since each batch constitutes a unique, patient-specific lot, any deviation in temperature during cryopreserved transit or a mix-up at the apheresis center can result in catastrophic, fatal graft-versus-host reactions. Furthermore, standardizing manufacturing processes across multiple international clinical sites remains hindered by inherent biological variability; the starting material, a patient’s own heavily pretreated and fundamentally exhausted immune cells, frequently yields inconsistent *ex vivo* expansion rates and variable ultimate product viability [154].

Cost and scalability represent the ultimate barriers to the widespread adoption of cell therapy in cervical cancer. The high incidence and mortality of cervical cancer are disproportionately concentrated in low- and middle-income countries [155]. However, the current infrastructural prerequisites for ACT, including state-of-the-art GMP facilities, highly specialized personnel, and intensive care unit capabilities for toxicity management, restrict these therapies to elite, high-resource academic medical centers [156].

Beyond manufacturing logistics, three interrelated challenges, cost-effectiveness, accessibility in low- and middle-income countries (LMICs), and real-world feasibility, remain underexplored. Current autologous TIL and CAR-T therapies carry price tags exceeding 400,000–600,000 per patient (excluding hospitalization and toxicity management), placing them far beyond conventional cost-effectiveness thresholds in most health systems. For cervical cancer, which disproportionately affects LMICs, no formal cost-effectiveness analyses have been published; extrapolation from melanoma and lymphoma suggests that even with breakthrough responses, the incremental cost-effectiveness ratio would likely exceed multiple GDP-per-capita benchmarks, precluding public funding. Over 90% of cervical cancer deaths occur in LMICs, yet these regions lack GMP facilities, trained apheresis teams, and intensive care units required for safe cell therapy. Even if products were donated, the absence of cold-chain infrastructure and specialized personnel would render delivery impossible. Allogeneic off-the-shelf platforms (e.g., CAR-NK, gene-edited universal T cells) could theoretically democratize access, but their per-dose cost remains uncertain, and regulatory pathways in LMICs are unprepared for such advanced therapies. In high-income settings, real-world adoption is hampered by prolonged vein-to-vein time (4–6 weeks for TILs), high rates of patient attrition during manufacturing, and the need for tertiary-care centres with 24/7 toxicity management. Broader implementation will require decentralized manufacturing (e.g., point-of-care closed systems), reduced lymphodepletion intensity, and cytokine-independent engineered cells. Until these barriers are addressed, cellular immunotherapy for cervical cancer will remain confined to a small fraction of eligible patients, exacerbating rather than reducing global oncological inequity.

To overcome these severe logistical and economic constraints, the clinical trial landscape is aggressively pivoting toward the development of allogeneic, “off-the-shelf” cellular therapies [157]. By utilizing healthy donor-derived NK cells (such as umbilical cord blood-derived NK cells) or utilizing gene-editing tools like CRISPR/Cas9 or TALENs to knock out the endogenous TCR and HLA molecules in healthy donor T cells (e.g., ALLO-316 targeting CD70), researchers aim to decouple the manufacturing process from the patient’s immediate clinical timeline [158]. These allogeneic approaches promise to dramatically reduce costs through batch manufacturing, enable immediate availability at the point of care, and democratize access to life-saving cellular immunotherapies for the global populations most heavily burdened by cervical cancer [159].

## 9 Author Perspective and Future Outlook

### 9.1 Author Perspective

Cellular immunotherapy is poised to fundamentally reshape the treatment paradigm for advanced cervical cancer, yet its maturation from a laboratory curiosity to a clinically accessible modality has been neither linear nor complete [140]. The past decade has witnessed transformative progress, driven by a deeper mechanistic understanding of HPV-driven immune evasion, the development of increasingly sophisticated engineering platforms, and the first durable complete responses in patients with refractory metastatic disease. Nevertheless, the path forward demands a clear-eyed appraisal of persistent obstacles and a strategic reorientation toward combinatorial, scalable, and safer next-generation approaches [160].

The central biological insight underpinning all current cellular strategies for cervical cancer is the viral aetiology of this malignancy [161]. Constitutive expression of the HPV E6 and E7 oncoproteins provides immunologically “non-self” antigens that are both necessary for tumor maintenance and absent from healthy tissues. This unique vulnerability has been most effectively exploited by tumor-infiltrating lymphocyte therapy, which has delivered unprecedented long-term remissions in a subset of heavily pretreated patients [162]. The FDA Breakthrough Therapy designation for lifileucel (LN-145) in cervical cancer, though distinct from full regulatory approval, which has been granted only for melanoma to date, marks a historic validation of the principle that autologous, polyclonal T cells can overcome the immunosuppressive fortress of cervical cancer. The FDA Breakthrough Therapy designation for lifileucel (LN-145) marks a historic validation of the principle that autologous, polyclonal T cells can overcome the immunosuppressive fortress of cervical cancer. However, the logistical complexity, prolonged vein-to-vein time, and dependence on a functionally intact autologous repertoire limit its scalability and generalizability. Moreover, even in the most optimized trials, a substantial fraction of patients fail to respond, highlighting the urgent need for predictive biomarkers and resistance-overcoming strategies [163].

TCR-engineered T cells offer a complementary, precision-engineered alternative. By redirecting peripheral blood T cells with high-affinity receptors against HPV16 E7 or E6 epitopes presented by specific HLA alleles, this platform bypasses the variability of endogenous TIL repertoires [130]. Clinical evidence suggests that E7-targeted TCR-T cells may be more potent than E6-directed constructs, a difference that likely reflects the relative stability and abundance of E7-derived peptides on tumor MHC molecules [164]. Nonetheless, HLA restriction remains an intrinsic barrier: each TCR is effective only in a subpopulation of patients carrying the relevant allotype, necessitating either a portfolio of allele-specific products or a radical re-engineering of antigen recognition logic. The emergence of “off-the-shelf” HLA-edited TCR-T cells from iPSC platforms represents an elegant solution, though proof-of-concept in cervical cancer remains preclinical.

Chimeric antigen receptor T cells, by contrast, operate independently of MHC presentation and can target surface-expressed tumor-associated antigens such as mesothelin, CD70, TROP2, and ALPP [165]. Their modular architecture has enabled successive generations of “armored” CARs that secrete cytokines (TRUCKs) or integrate JAK-STAT signaling domains, thereby enhancing persistence within the hostile solid TME. Yet the Achilles’ heel of CAR-T in cervical cancer is target selection: most surface antigens are not truly tumor-specific but are expressed at basal levels on vital epithelia, raising the specter of on-target, off-tumor toxicity that has proved fatal in early solid tumor trials. This has catalyzed the development of sophisticated safety switches (iCasp9) and logic-gated circuits (AND, NOT, OR gates) that confer spatial and temporal control, but clinical deployment of these synthetic biology tools is still nascent [166].

Among the four major platforms, CAR-NK cells have emerged as the most compelling candidate for an allogeneic, “off-the-shelf” therapy. Their innate capacity for missing-self recognition provides an intrinsic defense against MHC-downregulated tumor variants, while the favorable safety profile, characterized by minimal CRS and ICANS, obviates the need for intensive care infrastructure. Preclinical studies in cervical cancer have validated third-generation CAR-NK cells targeting FAP or mesothelin, and first-in-human trials of cord blood-derived TROP2-CAR-NK cells (NCT06066424) are now underway. The principal limitation remains limited *in vivo* persistence, which is being addressed by cytokine armoring (e.g., tethered IL-15) and by deriving NK cells from iPSCs that can be engineered for enhanced metabolic fitness. If these challenges are overcome, CAR-NK cells could democratize access to cellular therapy, particularly in low- and middle-income countries where the burden of cervical cancer is highest [167].

### 9.2 Future Outlook

Looking forward, the future of cellular therapy for cervical cancer will be defined not by competition among platforms but by their rational integration into combination regimens and by the resolution of manufacturing and safety barriers that currently restrict patient access [168]. First, combination with immune checkpoint blockade is already moving from empirical co-administration to engineered self-sufficiency: fourth-generation CARs that secrete anti-PD-L1 nanobodies locally within the tumor bed are entering clinical evaluation, potentially achieving synergistic efficacy without systemic toxicity. Second, the convergence of cellular therapy with conventional modalities, chemotherapy, radiotherapy, and targeted agents, offers a multi-pronged attack on the immunosuppressive TME. Immunogenic cell death induced by cisplatin or radiation can convert a “cold” tumor into a “hot” one, facilitating chemokine-driven infiltration of adoptively transferred cells. Third, epigenetic modulators such as EZH2 and BET inhibitors may upregulate surface antigen expression, increasing the avidity of CAR-T cells and circumventing antigen-loss escape [169,170].

On the safety front, the high frequency of low-grade CRS in cervical cancer trials, even with TILs, underscores the need for vigilant monitoring and protocolized use of tocilizumab. More fundamentally, the field must accelerate the clinical translation of logic-gated CARs and universal suicide switches to eliminate the risk of fatal off-tumor toxicity. Simultaneously, the preparative lymphodepletion regimen, currently a one-size-fits-all combination of fludarabine and cyclophosphamide, requires refinement. Biomarker-guided dosing, substitution with targeted lymphodepleting agents, or elimination of the most toxic components through cytokine-armored cellular products (e.g., IL-15-secreting CAR-NK cells) could substantially reduce the morbidity of bone marrow suppression and secondary infections [171,172].

Perhaps the most transformative shift will be the transition from autologous to allogeneic manufacturing. The current vein-to-vein time of four to six weeks is unacceptable for rapidly progressing metastatic cervical cancer, and the cost of bespoke GMP production places these therapies beyond reach for the majority of affected women worldwide. Allogeneic platforms, whether derived from umbilical cord blood, healthy donor peripheral blood, or iPSCs, promise to reduce costs by an order of magnitude, enable batch production and immediate availability, and decouple manufacturing from the patient’s clinical trajectory. Gene editing with CRISPR/Cas9 to disrupt endogenous TCR and HLA molecules can prevent graft-versus-host disease and host-versus-graft rejection, respectively, paving the way for truly universal “off-the-shelf” products [173].

## 10 Conclusions

In conclusion, cellular immunotherapy for cervical cancer stands at an inflection point. The scientific foundation has been solidly laid: we understand the viral antigens, the immunosuppressive landscape, and the engineering principles required to redirect immune cells. Early clinical proofs-of-concept have demonstrated that durable, even curative, responses are achievable in a subset of patients. The tasks for the coming decade are therefore not discovery-driven but translational and implementation-focused. They include: (i) expanding the responder population through rational combinations that remodel the TME; (ii) mitigating toxicity through synthetic biology safety devices; (iii) reducing cost and logistical burden through allogeneic, “off-the-shelf” products; and (iv) ensuring that these advances reach the low- and middle-income countries where cervical cancer continues to claim hundreds of thousands of lives each year. If these challenges are met, cellular therapy will move from a last-line salvage option to an integral component of front-line and adjuvant treatment for HPV-driven malignancies, fundamentally altering the natural history of a disease that has remained stubbornly lethal in its advanced stages.

## Data Availability

Not applicable.

## Abbreviations

The following abbreviations are used in this manuscript:

ACT	Adoptive Cell Therapy
ADCs	Antibody-Drug Conjugates
ALPP	Alkaline Phosphatase, Placental
BET	Bromodomain and Extra-Terminal motif
CAFs	Cancer-Associated Fibroblasts
CAR	Chimeric Antigen Receptor
CGT	Cell and Gene Therapy
CRISPR	Clustered Regularly Interspaced Short Palindromic Repeats
CRS	Cytokine Release Syndrome
CTL	Cytotoxic T Lymphocyte
DAMPs	Damage-Associated Molecular Patterns
DC	Dendritic Cell
EGFR	Epidermal Growth Factor Receptor
EZH2	Enhancer of Zeste Homolog 2
FAP	Fibroblast Activation Protein
FDA	Food and Drug Administration
FIGO	International Federation of Gynecology and Obstetrics
GAC	Gastric-type Endocervical Adenocarcinoma
GMP	Good Manufacturing Practice
HER2	Human Epidermal Growth Factor Receptor 2
HIF-1α	Hypoxia-Inducible Factor 1-alpha
HLA	Human Leukocyte Antigen
HPV	Human Papillomavirus
iCAFs	Inflammatory Cancer-Associated Fibroblasts
ICANS	Immune Effector Cell-Associated Neurotoxicity Syndrome
iCasp9	Inducible Caspase 9
ICD	Immunogenic Cell Death
IDO1	Indoleamine 2,3-Dioxygenase 1
IFN	Interferon
IL	Interleukin
iPSC	Induced Pluripotent Stem Cell
IRF-3	Interferon Regulatory Factor 3
JAK-STAT	Janus Kinase-Signal Transducer and Activator of Transcription
KLF2	Krüppel-Like Factor 2
LMICs	Low- and Middle-Income Countries
MDSCs	Myeloid-Derived Suppressor Cells
MeSH	Medical Subject Headings
MHC	Major Histocompatibility Complex
MSLN	Mesothelin
MUC1	Mucin 1
MUC16	Mucin 16
NCRs	Natural Cytotoxicity Receptors
NFAT	Nuclear Factor of Activated T cells
NK	Natural Killer
NKG2D	Natural Killer Group 2, member D
PDO	Patient-Derived Organoid
PD-1	Programmed Cell Death Protein 1
PD-L1	Programmed Death-Ligand 1
PLAP	Placental Alkaline Phosphatase
pRb	Retinoblastoma Protein
PSMA	Prostate-Specific Membrane Antigen
scFv	Single-Chain Variable Fragment
SPP1	Secreted Phosphoprotein 1
TALENs	Transcription Activator-Like Effector Nucleases
TAMs	Tumor-Associated Macrophages
TCR	T Cell Receptor
TGF-β	Transforming Growth Factor-beta
TILs	Tumor-Infiltrating Lymphocytes
TLR9	Toll-Like Receptor 9
TME	Tumor Microenvironment
TNF	Tumor Necrosis Factor
Tregs	Regulatory T Cells
TROP2	Trophoblast Cell Surface Protein 2
TRUCKs	T cells Redirected for Universal Cytokine-mediated Killing
α-SMA	Alpha-Smooth Muscle Actin
